# Foundation models in ophthalmology

**DOI:** 10.1136/bjo-2024-325459

**Published:** 2024-06-04

**Authors:** Mark A Chia, Fares Antaki, Yukun Zhou, Angus W Turner, Aaron Y Lee, Pearse A Keane

**Affiliations:** 1 Institute of Ophthalmology, University College London, London, UK; 2 NIHR Biomedical Research Centre, Moorfields Eye Hospital NHS Foundation Trust, London, UK; 3 The CHUM School of Artificial Intelligence in Healthcare, Montreal, Quebec, Canada; 4 Lions Outback Vision, Lions Eye Institute, Nedlands, Western Australia, Australia; 5 University of Western Australia, Perth, Western Australia, Australia; 6 Department of Ophthalmology, University of Washington, Seattle, Washington, USA; 7 Roger and Angie Karalis Johnson Retina Center, University of Washington, Seattle, Washington, USA

**Keywords:** Retina, Imaging

## Abstract

Foundation models represent a paradigm shift in artificial intelligence (AI), evolving from narrow models designed for specific tasks to versatile, generalisable models adaptable to a myriad of diverse applications. Ophthalmology as a specialty has the potential to act as an exemplar for other medical specialties, offering a blueprint for integrating foundation models broadly into clinical practice. This review hopes to serve as a roadmap for eyecare professionals seeking to better understand foundation models, while equipping readers with the tools to explore the use of foundation models in their own research and practice. We begin by outlining the key concepts and technological advances which have enabled the development of these models, providing an overview of novel training approaches and modern AI architectures. Next, we summarise existing literature on the topic of foundation models in ophthalmology, encompassing progress in vision foundation models, large language models and large multimodal models. Finally, we outline major challenges relating to privacy, bias and clinical validation, and propose key steps forward to maximise the benefit of this powerful technology.

## Introduction

 Over the past decade, there has been enormous interest in artificial intelligence (AI), both within healthcare and beyond. This has been primarily driven by advances in deep learning, a branch of AI that applies artificial neural networks to high-dimensional data to perform a range of complex tasks. Within medicine, ophthalmology has been at the forefront of these advances.^1^ Notable milestones include approval of the first two autonomous AI systems within medicine by the Food and Drug Administration,^2 3^ and the development of a comprehensive optical coherence tomography (OCT) triage system with expert-level performance.^4^ Perhaps of greatest significance have been applications which extend beyond ophthalmology, allowing the use of retinal imaging to derive insights into some of the most significant causes of death and disease globally.^5 6^ Despite this progress, the uptake of deep learning into real-world clinical use has been slow, hampered by challenges such as the need for robust clinical validation, regulatory approval, and integration with existing care and funding pathways.

Over the past year, interest in AI has skyrocketed to unprecedented levels, driven largely by the advent of so-called foundation models. To a larger extent than ever before, the extraordinary capabilities of AI have reached mainstream attention through the release of generative foundation models like ChatGPT and Stable Diffusion. We believe that as a specialty, ophthalmology remains well-placed to continue driving forward progress towards the applications of foundation models in healthcare. In particular, foundation models may offer solutions to some of the most significant implementation barriers, leading to transformative impacts on the care of sight-threatening eye conditions and major systemic diseases.

This review hopes to provide a roadmap for eyecare professionals on the potential of foundation models in ophthalmology, particularly for those interested in applying these advances to their own research and clinical practice. We begin by providing an overview of the key concepts underlying these models. Next, we summarise existing progress towards applying foundation models in the context of ophthalmology. Finally, we discuss barriers and future directions for ongoing progress in the field.

## What is a foundation model?

**Figure 1:**
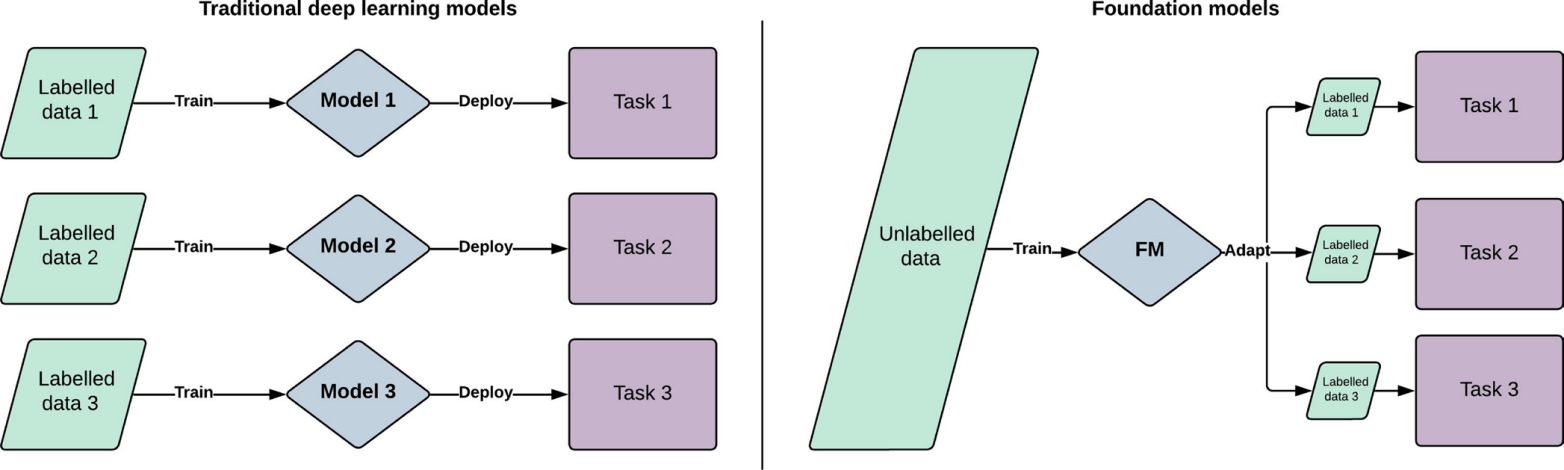
Schematic diagram comparing foundation models with traditional artificial intelligence models, showing the benefits of generalisability, label efficiency and computational efficiency. Rather than training a new model for each task, a single foundation model is generalisable to multiple downstream tasks. By learning general representation from vast quantities of unlabelled data, foundation models require less labelled data for each task (size of green boxes). These fine-tuning stages are also computationally efficient compared with training models from scratch. FM, foundation model.

The term foundation model was coined in 2021 by researchers at the Stanford University Institute for Human-Centred AI. It describes a large AI model trained on vast quantities of diverse data, which can then be adapted to a wide range of downstream tasks.^7^ Foundation model is a general term which can encapsulate models trained on a single modality such as text data (large language models, LLMs) or imaging data (large vision models), as well as models trained on multiple modalities such as vision language models (VLMs) and large multimodal models (LMMs). Although foundation models are based on standard deep learning and transfer learning techniques, they represent a fundamental change from traditional approaches, both in terms of their scale and intended scope.^7^ A comparison between these two approaches is outlined in figure 1. While previous generations of AI models were generally designed to solve single specific tasks, foundation models represent a versatile tool with potentially limitless applications. Their development has been enabled by larger datasets, novel training approaches and advances in model architecture.

Key advantages of foundation models include improved label efficiency, enhanced generalisability and reduced computational requirements during fine-tuning. Foundation models have the ability to learn universal patterns from data without specific labels, making them broadly useful for multiple tasks. Many of the properties of foundation models only develop once a critical threshold of scale is reached. This has been termed ‘emergent abilities’ and is one of the qualities which distinguish foundation models from traditional transfer learning.^8^ Due to the initial training at scale, a foundation model may require very few or even no labels when being adapted to a new task, referred to as few-shot and zero-shot learning, respectively. This enhanced label efficiency delivers the potential to design tasks targeted at rare diseases, even when little training data exists. Similarly, the ability to pretrain on diverse datasets can lead to improved performance on minority ethnic groups, which has been a key concern when attempting to implement models trained with traditional approaches. Finally, open-source foundation models can democratise access to AI and accelerate progress by circumventing the need for large datasets and extensive computational resources, which are major barriers to entry. Specific examples demonstrating how these advantages have been applied in the context of ophthalmology are outlined in subsequent sections.

## Self-supervised learning

The emergence of novel training approaches that can be applied to unlabelled data has been a key enabler for the development of foundation models. Traditional deep learning models are trained using supervised learning, whereby a model learns representations by mapping an input (eg, retinal photo) with a labelled output (eg, diagnosis of diabetic retinopathy).^9^ A supervised learning method therefore requires vast quantities of labelled data. Due to the requirement for specialised knowledge, labelling data in a medical context is time-consuming and expensive. Many of the major implementation challenges for deep learning models arise due to a paucity of diverse, labelled datasets. One approach to overcoming this problem is to initially train on natural (non-medical) image datasets, before performing transfer learning. While this does reduce label requirements, the solution is suboptimal due to the large differences between natural image datasets and medical datasets.^9^

**Figure 2:**
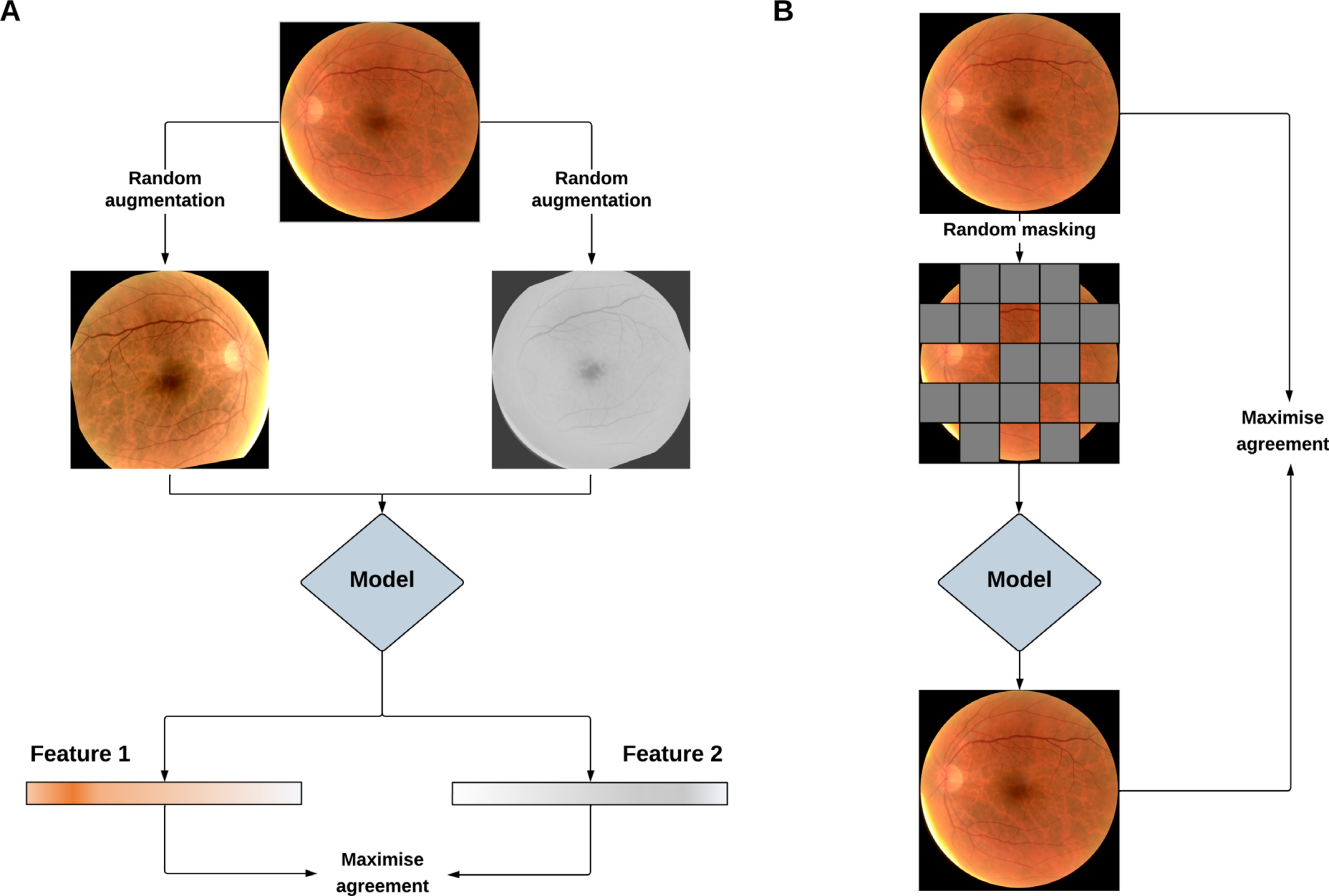
Pipeline for training vision foundation models using contrastive (A) and generative (B) self-supervised learning (SSL). In the contrastive SSL example, the pretext learning task involves applying random image augmentations and training a model to maximise the agreement of matching image pairs. In the generative SSL example, the pretext task involves masking areas of an image and training a model to reconstruct the missing portions. In both cases, the model learns general imaging features applicable to multiple downstream tasks.

In contrast to labelled datasets, unlabelled imaging data is ubiquitous in medicine, rapidly accumulating over the course of routine clinical care. For example, during 2022, almost 1.5 million images were acquired at Moorfields NHS Foundation Trust in London, UK. Self-supervised learning (SSL) provides the opportunity to tap into this vast quantity of unlabelled data which often goes unused. In the absence of labels, SSL representations by extracting labels from the data itself via a ‘pretext’ task. Pretext tasks can be broadly classified as being contrastive or generative in nature,^9^ as shown in figure 2. A contrastive approach generally involves augmenting the original images, such as through rotation or flipping. A model is then trained to maximise the similarity between augmented images from the matching originals, while separating those from non-matching originals. A generative approach usually involves discarding and generating image information, such as masking regions of an input image and then attempting to reconstruct the missing portions. An SSL approach that uses a well-chosen pretext task is a key component of developing a powerful foundation model that possesses robust and generalisable capabilities.

## Transformer architectures

Transformers are a type of neural network architecture that were originally described in 2017 when they were applied to natural language processing (NLP).^10^ They possess several distinct advantages compared with recurrent neural networks (RNNs), the dominant architecture used for NLP at the time. A key limitation of RNNs was that its structure required individual words to be processed sequentially, leading to poor scalability and limited contextual understanding. The transformer architecture addressed these barriers using two innovative approaches: positional encodings and attention mechanisms.^10^ Positional encodings allowed a network to understand the order of words by storing this information directly within the data itself, rather than relying on sequential processing as part of the network’s architecture. This structure led to drastic improvements in parallelisation—the ability to scale training to unprecedented levels by harnessing large datasets. Attention mechanisms, and in particular ‘self-attention’ were novel structures which allowed the network to better understand words in the context of surrounding words, thereby developing a robust internal representation of language. When combined with the enhanced availability of training data enabled by SSL, transformer architectures became a major driving force behind the enormous progress seen with LLMs in recent years.

A further key breakthrough occurred in 2020 when the transformer architecture was applied to imaging data in the form of vision transformers.^11^ The elegant approach involved partitioning an image into patches, followed by vectorisation with linear transformation. From this point, the image data could be treated in a similar way to text data, while still using positional encodings and attention mechanisms. This has led to the key benefit of transformers—the ability to capture global dependencies and contextual understanding in images using vast quantities of training data. Importantly, transformers afford greater flexibility, allowing the model to learn and adapt to patterns without being constrained by predetermined assumptions (inductive priors), as in the case of convolutional neural networks. Another strength of transformers arises from their universal structure, which enables flexible integration of different data types into a single model, such as text, language and audio data. This ability has paved the way for the development of VLMs and LMMs.

## Enhanced computer vision with foundation models

Despite the enormous potential for vision foundation models to revolutionise image-driven medical specialties, their application within ophthalmology remains relatively recent. In 2022, a Google research group introduced REMEDIS, a framework for building foundation models for medical imaging.^12^ The framework was used to create a suite of pretrained models for modalities across different specialties, including one for colour fundus photos. The approach used a combination of labelled and unlabelled fundus images in two stages: supervised learning on 300 million labelled natural images followed by contrastive self-supervised training on unlabelled fundus images. The pretrained model was then fine-tuned for the prediction of macular oedema in both an internal dataset, and an external dataset acquired on a different device and population.

The key findings showed that compared with a fully supervised approach, REMEDIS had better internal performance, a 93% reduction in label requirements when fine-tuned for the external dataset, as well as improved zero-shot external performance in two datasets with different ethnic distributions. Similar results were replicated for the other imaging modalities including chest X-rays and pathology slides. Although this work presented a strong initial framework for building models with better generalisability to ethnic groups and reduced training costs, the retinal image validation was limited to a single task. The key question of whether training on unlabelled retinal images could teach general representations applicable to diverse downstream tasks remained unanswered.

In 2023, our group released RETFound, a foundation model for retinal images.^13^ We trained RETFound sequentially on 1.3 million natural images followed by 1.6 million retinal images, both using a generative self-supervised technique called masked autoencoders.^14^ In this approach, 75% of the input image is masked and the model learns representations by attempting to reconstruct the missing patches. We then fine-tuned and validated RETFound on 13 downstream tasks across 2 modalities: OCT and retinal photography. The downstreams tasks varied considerably in scope and complexity, encompassing retinal disease diagnosis, retinal disease prediction, as well as prediction of future systemic events like myocardial infarction and stroke. Across these tasks we were able to demonstrate several key advantages of foundation models in comparison to competitive alternatives, including (1) improved internal performance, (2) improved zero-shot external performance, (3) better generalisability to ethnic subgroups, (4) enhanced label efficiency and (5) reduced computational requirements. In making RETFound openly available, we hope to democratise access to AI and accelerate progress towards implementing models that are generalisable and equitable.

Although the tasks explored in RETFound focus on classification of current or future disease, the training strategy used is likely also applicable to object detection and segmentation tasks. RETFound also separates OCTs and retinal photos into distinct models, despite there being potential advantages to developing a single foundation model which can flexibly integrate different imaging modalities. A number of preprints and brief reports have begun to explore segmentation tasks and multimodal integration in the context of ophthalmology, however work in this area remains limited.^15^,^17^

## Leveraging LLMs

LLMs are foundation models that are designed to understand and generate natural language.^18^ They are trained on vast corpora of text, including archives of the internet, books and encyclopaedias like Wikipedia.^19^ In that sense, once trained, LLMs contain a representation of the collective written knowledge of humanity until its training cut-off date.

**Figure 3:**
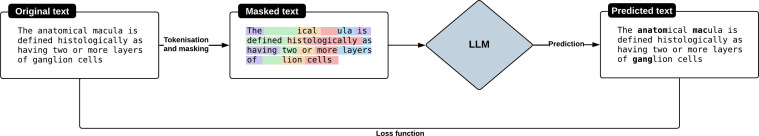
Pipeline for training a large language model. Text is separated into a series of tokens (coloured highlighting). A proportion of these tokens are masked, and the model is trained to predict these missing tokens via a loss function. LLM, large language model.

During training, LLMs process text as ‘tokens’ which are sequences of characters corresponding to words, parts of words or individual characters.^20^ LLMs learn to understand the statistical relationships between tokens as they appear in the training data, with the goal of predicting the next token in a sequence of tokens.^21^ After tokenisation, certain tokens are randomly masked, and the model is tasked to predict the original tokens based on the context provided by the remaining tokens.^21^ We illustrate an example in figure 3. This process is repeated at scale using billions to trillions of tokens.^19 22^ Once deployed, an LLM is prompted using natural language by the user, and it generates a response based on the statistical patterns it has learnt on the sequence of tokens.^23^

Before releasing LLMs to the public, developers typically undertake ‘alignment’ processes to mitigate the risk of generating inaccurate or harmful content and spreading misinformation.^24^ In general, it is agreed that they need to be ‘helpful, honest and harmless’.^24^ One way this can be achieved is through fine-tuning using reinforcement learning with human feedback.^25^ This is achieved by getting human evaluators to rank the outputs of the model, based on which a reward model is trained to assign scores to the model’s outputs. Reinforcement learning is then used to fine-tune the LLM, aiming to maximise these scores.^25^

In medicine, there has been growing interest in evaluating the usefulness of LLMs in encoding clinical knowledge.^26 27^ Both generalist all-purpose and medical fine-tuned models have been evaluated.^28 29^ In ophthalmology, most of the work has focused on evaluating generalist LLMs for their question-answering abilities.^30^,^32^ The performance of GPT-4 has been notably impressive, achieving a score of 72.9% on a multiple-choice question dataset, numerically surpassing the average historical human performance benchmarks.^31^ While those findings are noteworthy, the real challenge lies in demonstrating their clinical usefulness and effectively integrating them into the clinical decision-making process.^33^

Clinicians critically appraising LLM studies should be cognisant that LLM performance is intrinsically related to several factors: the content and formatting of the prompts used, which reflects how users interact with the model; the recency of the model’s training, indicating its currency and relevance; and the specific settings of the model, such as the temperature—a measure of the creativity of the output.^31 34^ LLM outputs should also be evaluated holistically, beyond accuracy or scores. To that extent, Singhal *et al* propose a framework for evaluating LLM answers in medicine.^26^ It includes the following elements: presence of incorrect information, agreement with scientific and clinical consensus, omission of content, extent and likelihood of harm, and bias in answers.

## Towards LMMs

**Figure 4:**
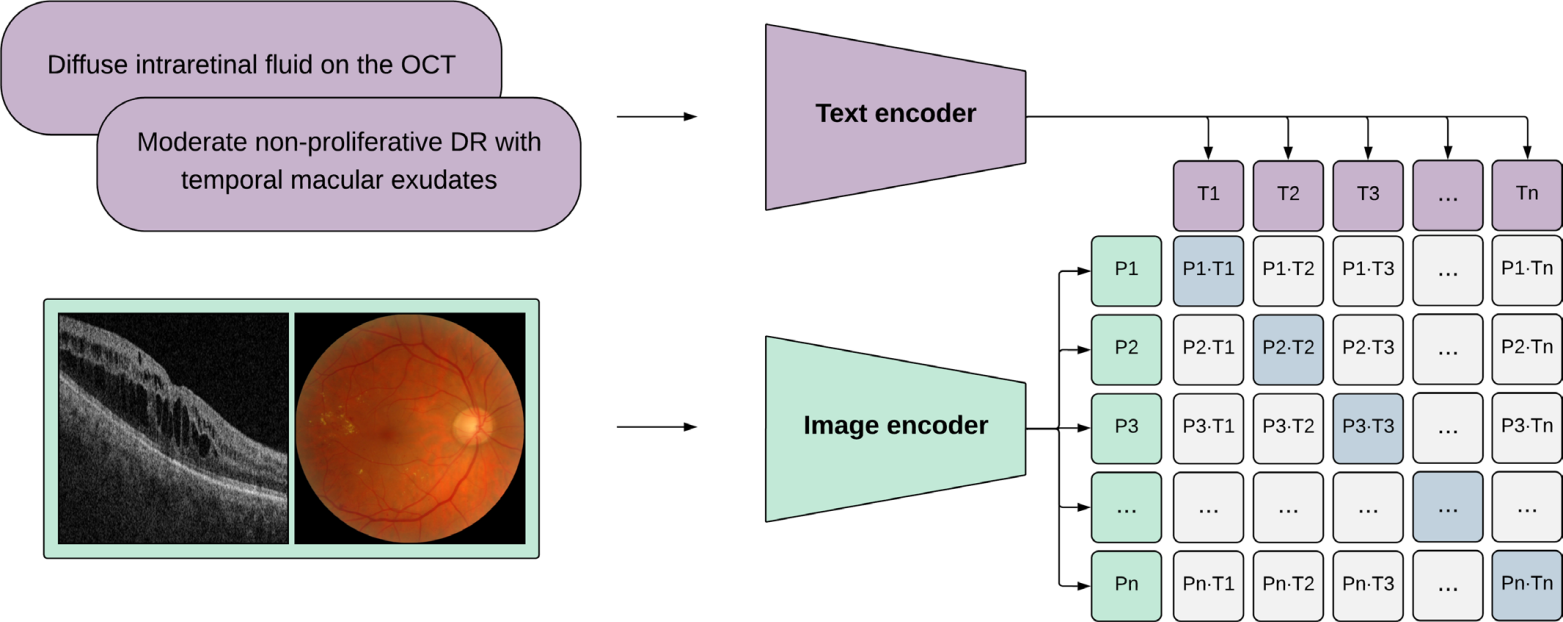
Pipeline for training vision-language models. The image and text data are independently processed by encoders to generate feature embeddings representative of images and text. The vision-language models are trained to maximise the agreement between image and text feature embeddings. The trained encoders apply to both image-based and text-based downstream tasks. OCT, optical coherence tomography; DR, diabetic retinopathy.

While text-based LLMs have shown significant potential in ophthalmology,^35^ models equipped with vision capabilities are poised to be the most beneficial. This reflects the inherent nature of ophthalmological practice, and our reliance on detailed visual examinations (supported by multimodal imaging) along with patient histories.^36 37^ Models such as Contrastive Language-Image Pre-training,^38^ which are capable of understanding images and text are also known as VLMs.^39^ Expanding on those capabilities, LMMs have been proposed to integrate ‘multisensory’ skills such as video, audio and sensor data.^40^ We show how VLMs can be trained in figure 4.

There is currently limited evidence on the performance of VLMs and LMMs in medicine and ophthalmology.^41^,^43^ Recent multimodal systems developed by Google have demonstrated early potential for LMMs to perform novel tasks such as visual question answering and report generation in the field of radiology. Med-PaLM Multimodal is a proof-of-concept generalist biomedical AI system that encodes and interprets multimodal data including language, imaging and genomics using the same set of model weights.^44^ For a sample of chest X-rays, clinicians preferred reports produced by Med-PaLM Multimodal over radiologists in 40% of cases. Another approach called ELIXR combined a fixed LLM with paired radiology images and reports, and was found to require two orders of magnitude less data to reach similar performance to a supervised contrastive learning approach.^45^

Building a multimodal model for ophthalmology from scratch faces the challenge of acquiring vast volumes of paired multimodal data, which is often scarce and costly to obtain due to the need for alignment and annotation. One potential solution is to leverage pre-existing vision foundation models and LLMs by integrating them into a multimodal framework, and subsequently fine-tuning the whole framework with a smaller quantity of paired data via transfer learning. Such a strategy has shown promising results in non-medical vision and language modelling.^46^ Another solution is to extend existing multimodal models in natural vision and language to medical fields via moderate transfer learning, as in the case of Med-PaLM which is based on PaLM-E.^47^

## Implications and challenges

Despite the enormous potential of foundation models in ophthalmology, addressing key challenges is crucial for their widespread adoption. While many of these challenges are pertinent to traditional deep learning approaches, the breadth of application for foundation models means that any harms may also be magnified.

Although RETFound showed improved performance in ethnic subgroups, the risk of bias from the underlying training data in foundation models persists. Previous studies have highlighted biases in AI models arising from under-representation in training data, or the reinforcement of harmful correlations.^48 49^ These biases could lead to poor performance in certain population groups, with a risk of perpetuating health inequities. The magnitude of training data required for foundation models may exacerbate this challenge, as evidence suggests that bias can increase with model scale.^50^ Mitigating this risk necessitates rigorous clinical validation and scrutiny of bias within training datasets. A significant stride in this direction is the establishment of standards for assessing diversity in health datasets, a primary goal of the STANDING Together initiative.^51^

The scale of training data also has implications for data privacy. In many cases, single institutions may struggle to amass sufficiently diverse datasets. There are numerous barriers to the development of foundation models which are particularly pertinent to low-resource settings. These include the significant cost of computational infrastructure, the development of streamlined pipelines for data curation, and the implementation of robust information governance processes. The integration of foundation models with privacy preserving techniques, such as federated learning, may facilitate collaborative training using data from multiple institutions, without the need for direct data access.^52^ While open-sourcing foundation models is crucial for maximising their benefits and accelerating progress, it must be balanced against associated privacy risks. Large models can have a tendency to memorise portions of training data and to repeat it to users,^53^ and models may be susceptible to malicious attacks aimed at extracting sensitive information.^54^

Finally, the enhanced generalisability of foundation models poses significant regulatory implications. For the safe implementation of a generalisable foundation model, it is crucial that these models express uncertainty when operating beyond the scope of their training data.^55^ Additionally, these models are likely to have heightened explainability requirements, such as the ability to reference evidence-based medicine sources.

## Future directions and potential

Foundation models in ophthalmology offer tremendous potential for transformative impact, opening up a variety of exciting research directions and applications within the field. Despite RETFound being trained on 1.6 million images, its model size remains relatively modest compared with many general-purpose language models. Expanding ophthalmic foundation models through increased data, parameters and advanced architectures represents a valuable next step. Scaling has proven to unlock novel capabilities in other contexts,^8 56^ and investigating these ‘emergent abilities’ within ophthalmology may unveil groundbreaking clinical applications.

Another compelling research avenue involves elevating the complexity and breadth of multimodal integration for foundation models. Striving towards truly multimodal foundation models with flexible human-AI interactions is a critical priority. This includes incorporating three-dimensional OCT data, seamlessly combining diverse imaging modalities and extending to true multimodality through the addition of functional tests, electronic health records, speech, text and genomic data. Achieving this comprehensive integration could lay the foundation for widespread applications in ophthalmology.^55^

**Figure 5:**
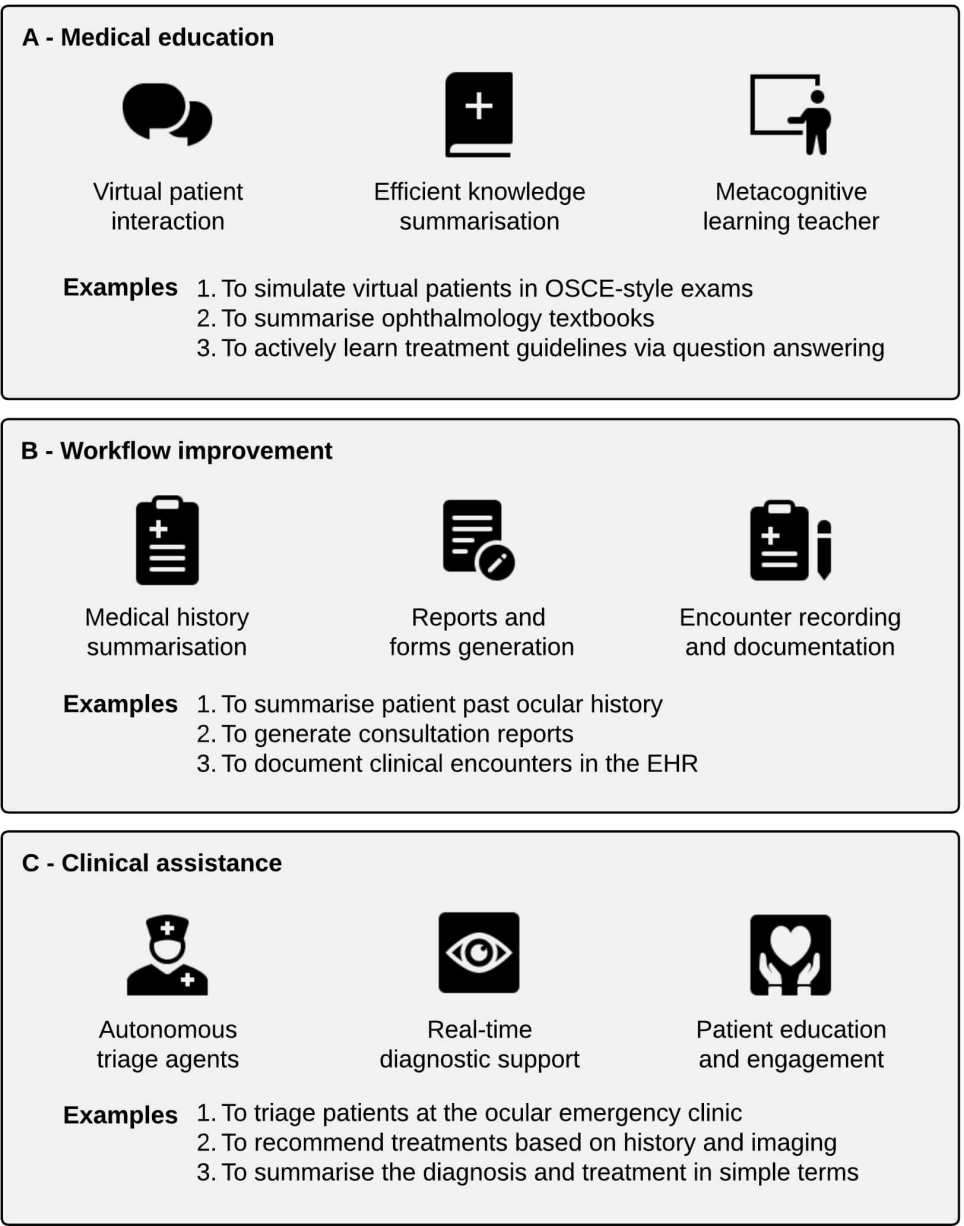
Overview of the applications of foundation models in ophthalmology. The most useful models for clinicians and patients are likely to be large multimodal models. Applications can be divided broadly into three categories: medical education (**A**), workflow improvement (**B**) and clinical assistance (**C**). EHR, electronic health record; OSCE, objective structured clinical examination.

Looking ahead, with the realisation of true multimodal capabilities, foundation models are poised to revolutionise various facets of ophthalmology. This encompasses contributions to medical education, optimisation of clinical workflows and direct clinical assistance at the bedside. Figure 5 outlines several proposed applications of foundation models in ophthalmology, showcasing the expansive and impactful possibilities.

## Conclusion

Foundation models signify a transformative leap, propelled by innovations such as SSL and transformer architectures. They hold immense potential to reshape clinical paradigms within ophthalmology, as evidenced by the remarkable strides in large vision and language models. As has been the case for other AI technologies, ophthalmology has the potential to act as an exemplar for other medical specialties by paving the way for the considered integration of foundation models into clinical care. It is critical that safety remains a prime consideration, with a focus on privacy protection, mitigation of bias and robust clinical validation. By embracing the advances brought by foundation models, balanced with safe and ethical practice, we can strive towards more equitable access to high-quality clinical care.

## Data Availability

Data sharing not applicable as no datasets generated and/or analysed for this study.
